# Efficacy and influencing factors analysis of SGLT2 inhibitors in treating heart failure following acute myocardial infarction

**DOI:** 10.3389/fphar.2025.1693420

**Published:** 2025-10-30

**Authors:** Haiping Du, Hui Xu, Jinwei Bao

**Affiliations:** Department of Cardiology, Yantaishan Hospital, Yantai, Shandong, China

**Keywords:** SGLT2 inhibitors, heart failure, acute myocardial infarction, LVEF improvement, NT-ProBNP, cardiovascular outcomes

## Abstract

**Purpose:**

Heart failure (HF) following acute myocardial infarction (AMI) significantly impacts morbidity and mortality. Sodium-glucose co-transporter 2 (SGLT2) inhibitors, initially developed for type 2 diabetes mellitus, have shown cardiovascular benefits. This study evaluates the efficacy of SGLT2 inhibitors in treating HF post-AMI compared to conventional treatments.

**Methods:**

We conducted a retrospective cohort study at our hospital from September 2022 to September 2024 involving 315 patients with HF post-AMI. Patients were categorized into a conventional treatment group (n = 140) and an SGLT2 inhibitor group (n = 175), with the latter further divided into effective (n = 154) and ineffective (n = 21) subgroups. Cardiac function was assessed pre- and post-treatment using echocardiography and serum biomarkers. Baseline characteristics and potential predictors of SGLT2 efficacy were also analyzed.

**Results:**

The SGLT2 group exhibited significant improvements in left ventricular ejection fraction (LVEF), decreased NT-proBNP, troponin I, and hs-CRP levels compared to the conventional group (*P* < 0.001). The overall effectiveness rate was 88.00% versus 75.71% in the conventional group (*P =* 0.004). Lower baseline LVEF and higher NT-proBNP levels were significant predictors of better outcomes. Notably, adverse reactions such as angina were reduced in the SGLT2 group.

**Conclusion:**

SGLT2 inhibitors were associated with enhanced cardiac function and reduce cardiac stress markers in HF patients post-AMI, suggesting their potential as an adjunctive therapy. Lower baseline LVEF and higher NT-proBNP levels may predict better response, suggesting their utility in personalized treatment strategies. This was a retrospective single-center study, and further prospective trials are needed to confirm these findings.

## 1 Introduction

Heart failure (HF) following acute myocardial infarction (AMI) represents a substantial burden on healthcare systems globally, accounting for significant morbidity and mortality (Akhtar et al., 2024). The pathophysiology of HF post-AMI was intricate and involves unfavorable cardiac remodeling, neurohormonal activation, and sustained myocardial inflammation, all of which contribute to the deterioration of cardiac function (Bertaina et al., 2023). Conventional therapeutic strategies, including beta-blockers, angiotensin receptor-neprilysin inhibitor (ARNI), and mineralocorticoid receptor antagonists (MRAs), have been cornerstones in managing HF post-AMI. However, despite these interventions, many patients remain at considerable risk of adverse cardiac events, necessitating exploration of novel therapeutic modalities (Bhasin et al., 2022).

In recent years, sodium-glucose co-transporter 2 (SGLT2) inhibitors have emerged as a promising pharmacological innovation initially targeted at glycemic control in type 2 diabetes mellitus (T2DM) (Aguilar-Gallardo et al., 2022). Intriguingly, SGLT2 inhibitors have demonstrated significant cardiovascular benefits, independent of their glucose-lowering effects, prompting extensive investigations into their utility across various cardiovascular conditions (Ali et al., 2024). These agents function primarily by reducing glucose and sodium reabsorption in the proximal renal tubules, leading to osmotic diuresis and natriuresis (Brito et al., 2020). This renal effect reduces intravascular volume and cardiac preload, thereby potentially ameliorating the hemodynamic burden on the heart—an effect particularly beneficial in the setting of HF (Cimino et al., 2022).

Several landmark trials, including the DAPA-HF and EMPEROR-Reduced, have underscored the efficacy of SGLT2 inhibitors in reducing HF hospitalizations and cardiovascular mortality (Goldman et al., 2023). These benefits extend to patients with HF with reduced ejection fraction (HFrEF) regardless of diabetes status, highlighting their potential to address the unmet needs in HF therapeutics (Hinton et al., 2021). However, the specific impact of SGLT2 inhibitors on patients with HF subsequent to AMI—a period marked by intense hemodynamic, metabolic, and inflammatory stressors—remains a field worthy of focused research (Hoehlschen et al., 2023).

The underlying mechanisms by which SGLT2 inhibitors confer cardioprotective effects were believed to be multifaceted (Koufakis et al., 2023). Beyond volume modulation, these agents appear to influence cardiac metabolism by promoting a shift from glucose oxidation to ketone body metabolism, which was more energy-efficient and potentially beneficial in a failing heart (Lahoti et al., 2021). Additionally, emerging evidence suggests that SGLT2 inhibitors may exert anti-inflammatory and antifibrotic effects, further mitigating pathogenic cardiac remodeling post-AMI (Liang and Gu, 2022).

Despite the promising data, individual patient responses to SGLT2 inhibitors can vary, influenced by baseline cardiac function. Therefore, understanding the factors that affect the efficacy of SGLT2 inhibitors in the context of HF post-AMI was critical for optimizing therapeutic outcomes and guiding personalized treatment strategies (Liang et al., 2023). Identifying these predictors can assist clinicians in stratifying patients who were most likely to benefit from SGLT2 inhibitor therapy, thereby enhancing the overall effectiveness of the intervention.

This study aims to evaluate the efficacy of SGLT2 inhibitors in patients with HF following AMI, comparing outcomes to a conventional treatment cohort. Furthermore, it seeks to identify factors that influence treatment efficacy, providing insights into which patient characteristics correlate with improved outcomes.

## 2 Materials and methods

### 2.1 Study design

This research employed a retrospective cohort study design to investigate patients with HF following AMI, treated at our hospital from September 2022 to September 2024. Based on their treatment modalities, patients were categorized into two groups: the conventional treatment group (n = 140) and the SGLT2 inhibitor group (n = 175). Additionally, the SGLT2 inhibitor group was subdivided into an effective group (n = 154) and an ineffective group (n = 21), depending on the efficacy of the treatment.

The Institutional Review Board and Ethics Committee of our institution approved this study. The requirement for informed consent was waived due to the retrospective nature of the study and exclusive reliance on de-identified patient data. Since this approach posed no potential risk or impact on patient care, the waiver was granted in accordance with the relevant regulatory and ethical guidelines governing retrospective research.

### 2.2 Eligibility and grouping criteria

Inclusion criteria: participants were included if they met the diagnostic criteria for AMI and HF (Zeymer et al., 2020; Ostrominski et al., 2024), had heart function classified as New York Heart Association (NYHA) Class II to IV, and were admitted to the hospital for treatment within 12 h of the AMI onset, provided their vital signs were stable. Additional criteria included being aged 18 years or older, possessing normal cognitive and mental status to ensure the reliability of symptom reporting and medical history documented in the records, having complete clinical documentation, and being first-time recipients of the relevant treatments.

Exclusion criteria: individuals were excluded if they had a pre-existing diagnosis of HF; exhibited cardiogenic shock, severe valvular disease, severe infection, or malignant tumors; suffered from severe organ dysfunction, specifically of the liver, gallbladder, or kidneys; had a history of cardiac surgery; had a known allergy to the medications related to the study; or were lactating or pregnant women.

### 2.3 Treatment approach

In this retrospective study, the assignment to treatment groups was based on the clinical decisions of the attending physicians, not on randomization. Both groups received fundamental treatments, which included oxygen therapy, antiplatelet agents, β-blockers, vasodilators, and antifibrotic therapy. The control group was administered Entresto (Sacubitril/Valsartan, Approval Number: HJ20170362, Novartis Singapore Pharmaceutical Manufacturing Private Ltd., Singapore, Specification: 50 mg). The initial dose was set at 50 mg taken twice daily. Over a period of 3 months, the dose was progressively increased to a target of 100 mg twice daily, guided by the same protocol of patient tolerance, blood pressure, and renal function for both groups. Treatment adherence was assessed retrospectively via pharmacy dispensing records and documentation in clinical notes, and was found to be comparably high in both groups.

Meanwhile, the SGLT2 inhibitor group received dapagliflozin (Approval Number: HJ20170119, AstraZeneca Pharmaceutical Co., Ltd., United Kingdom, Specification: 10 mg) in addition to the basic treatments. This group commenced with an initial dose of 5 mg once daily, which was gradually increased to 10 mg once daily. The duration of this treatment was also 3 months.

### 2.4 General information

Patient demographic information, including age, sex, and body mass index (BMI), as well as NYHA classification and disease-related characteristics such as heart rate, blood pressure, and medication use, were obtained from the medical record system. Additionally, any adverse reactions occurring during the treatment period were documented.

The New York Heart Association (NYHA) functional classification system was the most widely used method for assessing heart function. This system classifies patients into four categories based on the symptoms and exercise capacity related to HF. Class I indicates an absence of HF symptoms, while symptoms become progressively more severe from Class II through Class IV.

### 2.5 Echocardiography examination

A color Doppler echocardiography system (Vivid E95, GE, United States) was employed to assess the left ventricular ejection fraction (LVEF) using a 3.0 MHz probe frequency, both prior to and following treatment. Subjects were positioned in the left lateral position to acquire the apical four-chamber view for pulsed Doppler measurements. Measurements of the left ventricular end-diastolic dimension (LVEDD), left ventricular end-systolic dimension (LVESD), and left ventricular end-diastolic volume (LVEDV) were captured via the parasternal long-axis view. The left ventricular mass (LVM) was calculated using the area-length method, from which the left ventricular mass index (LVMI) was derived. The left ventricular remodeling index (LVRI) was calculated by dividing the LVM by the LVEDV.

### 2.6 Blood test

Fasting venous blood samples (5 mL) were collected from patients before and after treatment. These samples were anticoagulated using ethylenediaminetetraacetic acid and centrifuged at 2,800 rpm for 15 min to separate the serum layer. Leukocytes and neutrophils count were performed using an automated hematology analyzer (BC-5000, Mindray, China). The levels of N-terminal pro-brain natriuretic peptide (NT-proBNP) were measured using an enzyme-linked immunosorbent assay (ELISA) (ab263877, Abcam plc, United Kingdom), while troponin I, high-sensitivity C-reactive protein (hs-CRP) and interleukin-6 (IL-6) levels were determined with an automated biochemical analyzer (BS-280, Mindray, China).

### 2.7 Efficacy evaluation

In accordance with relevant guidelines (Rogers and Bush, 2015), the effectiveness of treatment for both groups was evaluated post-treatment. The criteria for determining effectiveness were defined as follows:

Significantly effective: substantial relief of clinical symptoms accompanied by an improvement of at least two classes in heart function.

Effective: partial relief of clinical symptoms with an improvement of one class in heart function.

### 2.8 Ineffective: no relief of clinical symptoms and no change in the heart function classification

Given the retrospective nature of this study, blinded assessment was not feasible. However, the classification was primarily based on the objective change in NYHA functional class, supplemented by documented clinical symptom relief from the medical records, to ensure consistency and minimize assessment bias.

### 2.9 Statistical analysis

The data were analyzed using SPSS 29.0 statistical software (SPSS Inc., Chicago, IL, United States). Categorical data were presented as [n (%)]. The chi-square test was applied using the basic formula for sample sizes ≥40 and theoretical frequencies T ≥ 5, with χ^2^ as the test statistic. When the sample size was ≥40 and theoretical frequencies were 1 ≤ T < 5, the chi-square test was adjusted with a correction formula. For sample sizes <40 or when theoretical frequency T < 1, Fisher’s exact test was used.

Continuous variables were first assessed for normal distribution using the Shapiro-Wilk test. Normally distributed continuous data were reported as means ± standard deviation (X ± s). Non-normally distributed data were analyzed with the Wilcoxon rank-sum test and presented as [median (25% quantile, 75% quantile)]. A p-value of less than 0.05 was considered statistically significant. Correlation analysis was conducted using Pearson correlation for continuous variables and Spearman correlation for categorical variables. Variables showing significant differences in both difference analysis and correlation analysis were included as covariates in the logistic regression analysis.

## 3 Results

### 3.1 Demographic and basic data

The mean age was 65.13 ± 5.16 years in the conventional group and 64.53 ± 5.41 years in the SGLT2 group (*P =* 0.314) (Table 1). Gender distribution was similar, with males comprising 36.43% and 32.57% of the conventional and SGLT2 groups, respectively (*P =* 0.474). Body mass index and smoking history were also akin between the two groups, with P Values of 0.078 and 0.392. Hypertension, diabetes, history of MI, atrial fibrillation, and NYHA grades showed no significant differences (all *P* > 0.05). Vital signs, including heart rate, systolic and diastolic pressures, and the use of ARNI, MRAs, and β-blockers were comparable, with P Values indicating no significant variance (all *P* > 0.05). These data suggest that the baseline characteristics of the two groups were well matched, eliminating potential confounding variables in evaluating the efficacy of SGLT2 inhibitors in treating HF following AMI.

**TABLE 1:** Demographic and basic data.

Parameters	Conventional treatment group (n = 140)	SGLT2 inhibitor group (n = 175)	t/χ^2^	P
Age (years)	65.13 ± 5.16	64.53 ± 5.41	1.008	0.314
Gender (Male, %)	51 (36.43%)	57 (32.57%)	0.514	0.474
BMI (kg/m^2^)	26.26 ± 2.32	26.71 ± 2.18	1.769	0.078
Hypertension (%)	88 (62.86%)	107 (61.14%)	0.097	0.756
Diabetes (%)	49 (35.00%)	67 (38.29%)	0.361	0.548
Smoking history (%)	57 (40.71%)	63 (36.00%)	0.733	0.392
Drinking history (%)	15 (10.71%)	21 (12.00%)	0.127	0.722
NYHA grade (%)			2.160	0.340
-Ⅱ	38 (27.14%)	56 (32.00%)		
-Ⅲ	62 (44.29%)	81 (46.29%)		
-Ⅳ	40 (28.57%)	38 (21.71%)		
History of MI (%)	5 (3.57%)	8 (4.57%)	0.197	0.658
Atrial fibrillation (%)	19 (13.57%)	25 (14.29%)	0.033	0.856
Heart rate (bpm)	81.78 ± 14.36	81.67 ± 14.23	0.070	0.944
Systolic pressure (mmHg)	125.71 ± 24.13	126.32 ± 24.26	0.223	0.824
Diastolic pressures (mmHg)	78.25 ± 14.53	79.35 ± 14.21	0.676	0.499
Use of ARNI (sacubitril/valsartan) (%)	94 (67.14%)	114 (65.14%)	0.139	0.710
Use of MRA (%)	67 (47.86%)	79 (45.14%)	0.230	0.631
Use of β-blockers (%)	119 (85.00%)	154 (88.00%)	0.606	0.436

BMI, body mass index; NYHA, new york heart association; MI, myocardial infarction; ARNI, angiotensin receptor-neprilysin inhibitor; MRA, mineralocorticoid receptor antagonist; SGLT2, sodium-dependent glucose transporters 2.

### 3.2 Comparison of indexes related to cardiac function between the two groups

**FIGURE 1:**
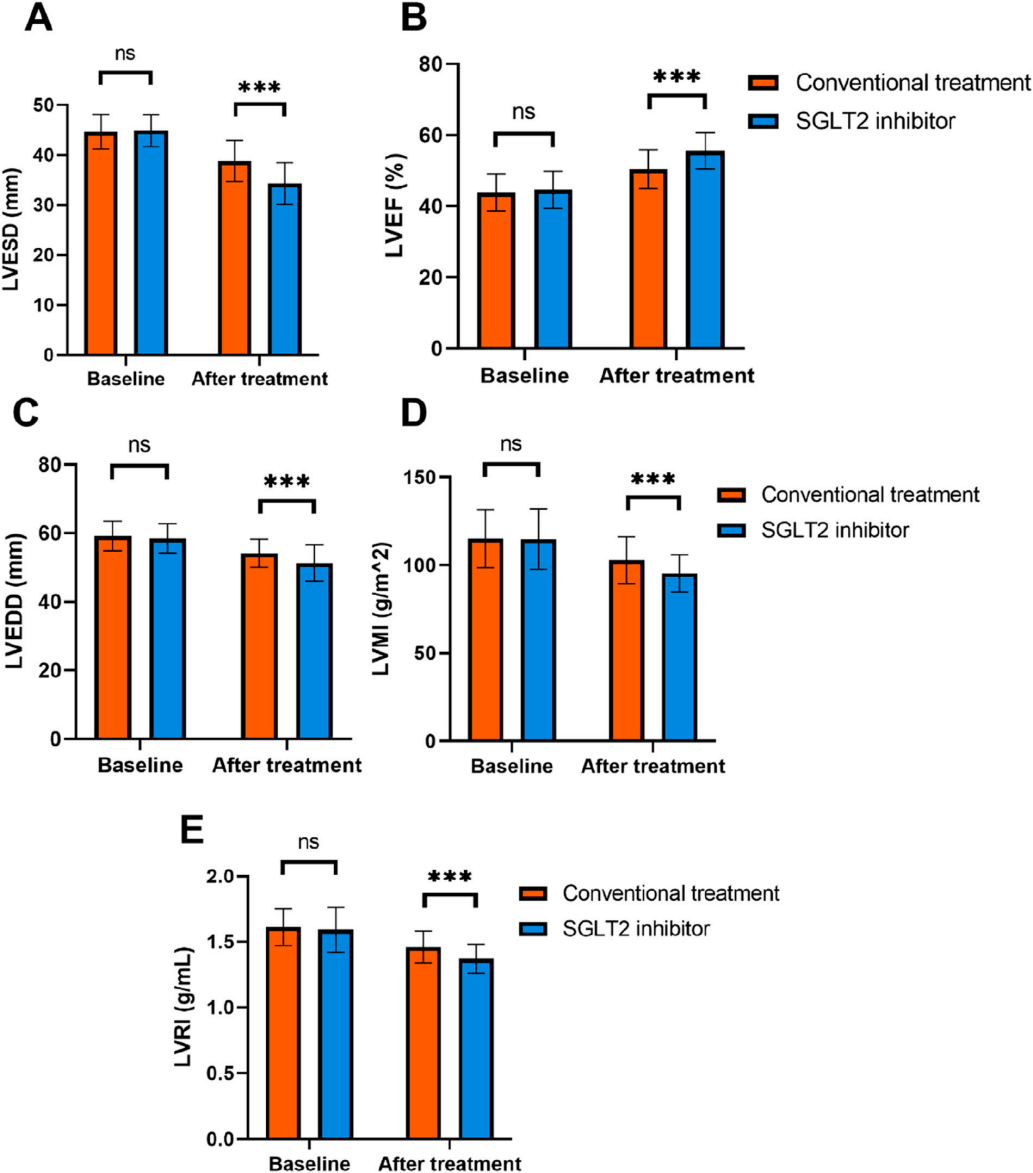
Comparison of indexes related to cardiac function between the two groups. **(A)** LVESD; **(B)** LVEF; **(C)** LVEDD; **(D)** LVMI; **(E)** LVRI. Note: SGLT2, sodium-dependent glucose transporters 2; LVEF, Left ventricular ejection fraction; LVESD, Left ventricular end-systolic dimension; LVEDD, Left ventricular end-diastolic dimension; LVMI, Left ventricular mass index; LVRI, Left ventricular remodeling index; ns, not significant; ***P < 0.001.

At baseline, there were no significant differences between the groups in LVESD, LVEF, LVEDD, left ventricular mass index (LVMI), and left ventricular remodeling index (LVRI) (*P* > 0.05) (Figure 1). However, after treatment, the SGLT2 inhibitor group showed significantly greater improvement in LVESD, reduced from 44.89 ± 3.21 mm to 34.32 ± 4.15 mm, compared to 38.83 ± 4.11 mm in the conventional group (*P* < 0.001). Similarly, LVEF increased from 44.59% ± 5.23% to 55.56% ± 5.16% in the SGLT2 group, while the conventional group achieved an increase to 50.38% ± 5.47% (*P* < 0.001). Improvements were also observed in LVEDD, reduced to 51.32 ± 5.29 mm in the SGLT2 group from a baseline of 58.48 ± 4.26 mm, significantly outperforming the conventional group (54.16 ± 4.13 mm post-treatment, *P* < 0.001). The SGLT2 group also showed greater reductions in LVMI and LVRI, with post-treatment values of 95.36 ± 10.49 g/m^2^ and 1.37 ± 0.11 g/mL, respectively, both demonstrating significant differences compared to the conventional group (*P* < 0.001). These results indicate that SGLT2 inhibitors substantially enhance cardiac function in HF patients post- AMI.

### 3.3 Comparison of circulating cardiac biomarkers and inflammatory biomarkers between the two groups

**FIGURE 2:**
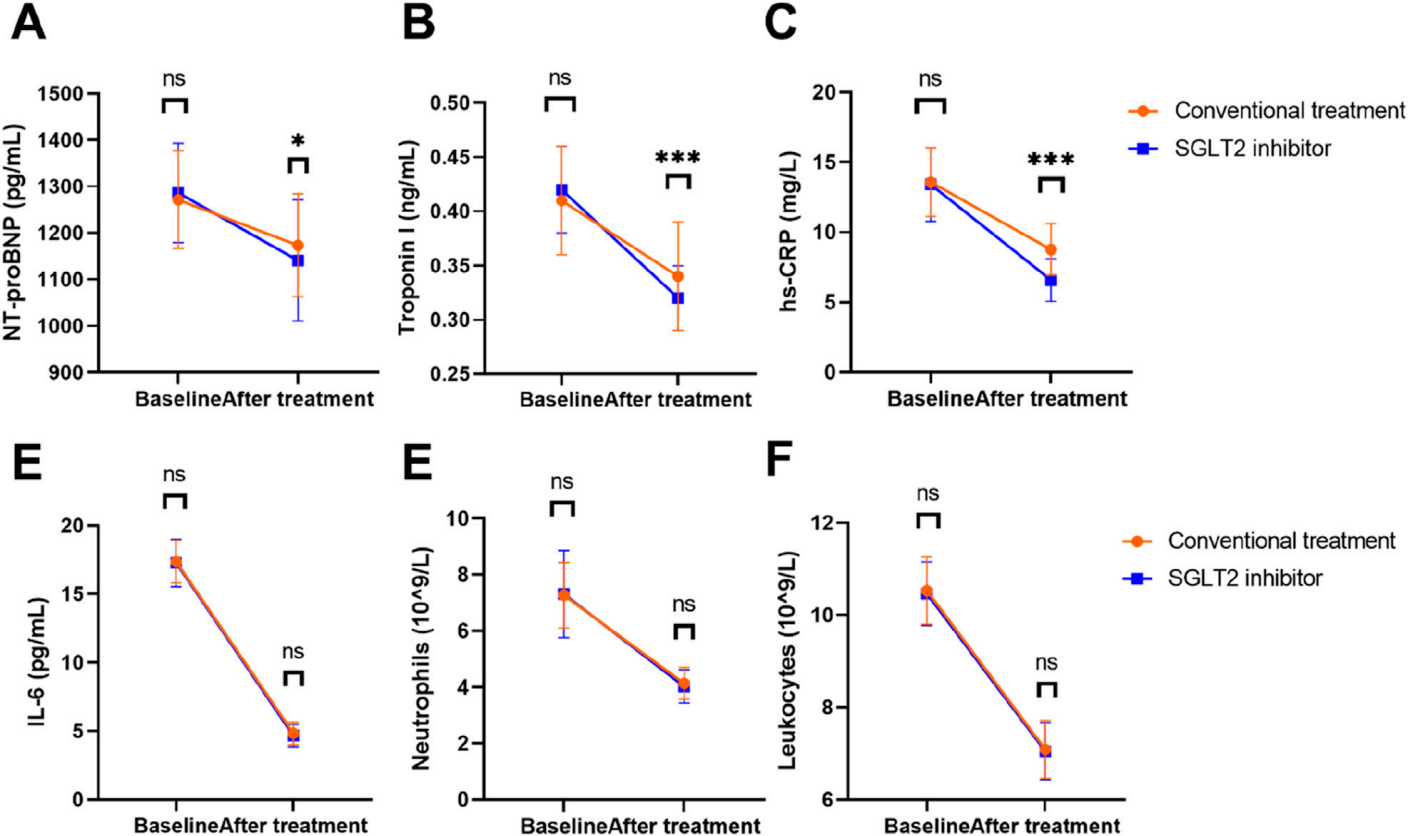
Comparison of circulating cardiac biomarkers and inflammatory biomarkers between the two groups. **(A)** NT-proBNP; **(B)** troponin I; **(C)** hs-CRP; **(D)** IL-6; **(E)** neutrophils; **(F)** leukocytes. Note: SGLT2, sodium-dependent glucose transporters 2; NT-proBNP, N-terminal prohormone of brain natriuretic peptide; hs-CRP, High-sensitivity C-reactive protein; IL-6, interleukin-6. ns, not significant; *, *P* < 0.05; ***P < 0.001.

At baseline, there were no significant differences in NT-proBNP levels (*P =* 0.218), troponin I levels (*P =* 0.129), hs-CRP levels (*P =* 0.550), IL-6 levels (*P =* 0.614), neutrophils (*P =* 0.795) and leukocyte (*P =* 0.374) between the two groups (Figure 2). However, following treatment, the SGLT2 inhibitor group demonstrated a significant reduction in NT-proBNP levels to 1,140.83 ± 130.21 pg/mL, compared to 1,173.12 ± 110.56 pg/mL in the conventional group (*P =* 0.018). Troponin I levels also decreased significantly in the SGLT2 group, reaching 0.32 ± 0.03 ng/mL versus 0.34 ± 0.05 ng/mL in the control group (*P* < 0.001). Moreover, hs-CRP levels were markedly lower in the SGLT2 inhibitor group post-treatment, at 6.58 ± 1.53 mg/L, compared to 8.75 ± 1.85 mg/L in the conventional treatment group (*P* < 0.001). There was no statistically significant difference in IL-6 levels, neutrophils and leukocytes between the two groups after treatment (all P > 0.05).

### 3.4 Comparison of adverse reactions between the two groups

Incidences of nausea (5.00% vs. 7.43%, *P =* 0.380), dizziness (0.71% vs. 2.29%, *P =* 0.512), abnormal renal function (1.43% vs. 2.29%, *P =* 0.890), hypotension (2.14% vs. 1.71%, *P =* 1.000), and hypoglycemia (2.86% vs. 3.43%, *P =* 1.000) were comparable between the groups (Table 2). However, the incidence of angina pectoris was significantly lower in the SGLT2 inhibitor group (4.00%) compared to the conventional treatment group (12.86%) (*P =* 0.004). These findings indicate that while most adverse reactions were similar across the groups, the SGLT2 inhibitors were associated with a reduced incidence of angina pectoris in patients with HF following AMI.

**TABLE 2:** Comparison of adverse reactions between the two groups.

Parameters	Conventional treatment group (n = 140)	SGLT2 inhibitor group (n = 175)	χ^2^	P
Nausea (%)	7 (5.00%)	13 (7.43%)	0.771	0.380
Dizzy (%)	1 (0.71%)	4 (2.29%)	0.429	0.512
Abnormal renal function (%)	2 (1.43%)	4 (2.29%)	0.019	0.890
Angina pectoris (%)	18 (12.86%)	7 (4.00%)	8.351	0.004
Hypotension (%)	3 (2.14%)	3 (1.71%)	0.000	1.000
Hypoglycemia (%)	4 (2.86%)	6 (3.43%)	0.000	1.000

SGLT2, sodium-dependent glucose transporters 2.

### 3.5 Efficacy evaluation

In the SGLT2 inhibitor group, 62.86% of patients experienced a significant effective response versus 38.57% in the conventional group (Table 3). Additionally, the effective response rate was 25.14% in the SGLT2 group compared to 37.14% in the conventional group. The rate of ineffective treatment was lower in the SGLT2 group at 12.00%, compared to 24.29% in the conventional treatment group. The overall effectiveness rate was significantly higher in the SGLT2 inhibitor group, with 88.00% of patients experiencing a positive response, as opposed to 75.71% in the conventional group (χ^2^ = 8.146, *P =* 0.004). These results indicate that SGLT2 inhibitors were more effective in treating HF following AMI.

**TABLE 3:** Efficacy evaluation.

Parameters	Conventional treatment group (n = 140)	SGLT2 inhibitor group (n = 175)	χ^2^	P
Significant effective	54 (38.57%)	110 (62.86%)		
Effective	52 (37.14%)	44 (25.14%)		
Ineffective	34 (24.29%)	21 (12.00%)		
Overall effectiveness rate (%)	106 (75.71%)	154 (88.00%)	8.146	0.004

SGLT2, sodium-dependent glucose transporters 2.

### 3.6 General information of patients using SGLT2 inhibitors

The average age was similar between the effective (64.63 ± 5.55 years) and ineffective groups (63.78 ± 4.30 years) (*P =* 0.501) (Table 4). Gender distribution showed a higher proportion of males in the ineffective group (47.62%) compared to the effective group (30.52%), though this difference was not statistically significant (*P =* 0.117). Other demographics and clinical characteristics, including BMI, presence of hypertension, diabetes, smoking, and drinking history, also showed no significant differences (*P* > 0.05). The New York Heart Association (NYHA) grades and history of myocardial infarction were comparable between groups. Heart rate and blood pressure measurements did not differ significantly, with P Values indicating no statistical significance (*P* > 0.05). Medication use, such as ARNI, MRAs, and β-blockers, was similar between the two groups (*P* > 0.05). These findings suggest that traditional demographic and clinical parameters did not significantly predict the efficacy of SGLT2 inhibitors in treating HF following AMI.

**TABLE 4:** General information of patients using SGLT2 inhibitors.

Parameters	Effective group (n = 154)	Ineffective group (n = 21)	t/χ^2^/fisher	P
Age (years)	64.63 ± 5.55	63.78 ± 4.30	0.675	0.501
Gender (Male, %)	47 (30.52%)	10 (47.62%)	2.460	0.117
BMI (kg/m^2^)	26.65 ± 2.23	27.19 ± 1.68	1.061	0.290
Hypertension (%)	96 (62.34%)	11 (52.38%)	0.771	0.380
Diabetes (%)	57 (37.01%)	10 (47.62%)	0.880	0.348
Smoking history (%)	58 (37.66%)	5 (23.81%)	1.539	0.215
Drinking history (%)	18 (11.69%)	3 (14.29%)	0.000	1.000
NYHA grade (%)			0.122	0.941
-Ⅱ	49 (31.82%)	7 (33.33%)		
-Ⅲ	72 (46.75%)	9 (42.86%)		
-Ⅳ	33 (21.43%)	5 (23.81%)		
History of MI (%)	7 (4.55%)	1 (4.76%)	None	1.000
Atrial fibrillation (%)	20 (12.99%)	5 (23.81%)	0.994	0.319
Heart rate (bpm)	81.61 ± 14.14	82.09 ± 15.18	0.144	0.886
Systolic pressure (mmHg)	126.45 ± 23.77	125.32 ± 28.21	0.200	0.842
Diastolic pressures (mmHg)	79.93 ± 14.11	75.11 ± 14.61	1.464	0.145
Use of ARNI (sacubitril/valsartan) (%)	99 (64.29%)	15 (71.43%)	0.415	0.519
Use of MRA (%)	67 (43.51%)	12 (57.14%)	1.388	0.239
Use of β-blockers (%)	138 (89.61%)	16 (76.19%)	2.009	0.156

BMI, body mass index; NYHA, new york heart association; MI, myocardial infarction; ARNI, angiotensin receptor-neprilysin inhibitor; MRA, mineralocorticoid receptor antagonist; SGLT2, sodium-dependent glucose transporters 2.

### 3.7 Baseline parameters of patients using SGLT2 inhibitors

The effective group exhibited a higher baseline LVEF at 45.13% ± 5.09%, compared to 40.64% ± 4.64% in the ineffective group (*P* < 0.001), indicating a potential influence on treatment efficacy (*P* > 0.05) (Table 5). Additionally, NT-proBNP levels were significantly lower in the effective group (1,277.05 ± 101.48 pg/mL) compared to the ineffective group (1,352.64 ± 122.73 pg/mL) (*P =* 0.002), suggesting a correlation between baseline cardiac stress markers and treatment outcomes. Other parameters, including LVESD, LVEDD, LVMI, LVRI, troponin I, hs-CRP, IL-6, Neutrophils and Leukocytes did not show statistically significant differences between the groups (*P* > 0.05). These results highlight the importance of baseline LVEF and NT-proBNP levels in predicting the efficacy of SGLT2 inhibitors in HF management post- AMI.

**TABLE 5:** Baseline parameters of patients using SGLT2 inhibitors.

Parameters	Effective group (n = 154)	Ineffective group (n = 21)	t	P
LVESD (mm)	44.93 ± 3.17	44.61 ± 3.54	0.430	0.667
LVEF (%)	45.13 ± 5.09	40.64 ± 4.64	3.825	<0.001
LVEDD (mm)	58.36 ± 4.24	59.35 ± 4.42	0.996	0.321
LVMI (g/m^2^)	115.02 ± 16.87	113.39 ± 19.50	0.407	0.684
LVRI (g/mL)	1.59 ± 0.17	1.60 ± 0.17	0.382	0.703
NT-proBNP (pg/mL)	1,277.05 ± 101.48	1,352.64 ± 122.73	3.120	0.002
Troponin I (ng/mL)	0.42 ± 0.04	0.42 ± 0.04	0.042	0.967
hs-CRP (mg/L)	13.34 ± 2.59	13.87 ± 3.03	0.864	0.389
IL-6 (pg/mL)	17.11 ± 1.35	17.47 ± 1.17	1.176	0.241
Neutrophils (10^9^/L)	7.24 ± 1.06	7.64 ± 1.16	1.594	0.113
Leukocytes (10^9^/L)	10.34 ± 0.77	10.53 ± 0.64	1.124	0.263

SGLT2, sodium-dependent glucose transporters 2; LVEF, left ventricular ejection fraction; LVESD, left ventricular end-systolic dimension; LVEDD, left ventricular end-diastolic dimension; LVMI, left ventricular mass index; LVRI, left ventricular remodeling index; NT-proBNP, N-terminal prohormone of brain natriuretic peptide; hs-CRP, high-sensitivity C-reactive protein; IL-6, interleukin-6.

### 3.8 Correlation analysis between SGLT2 inhibitors efficacy and various factors

The correlation analysis revealed significant relationships between the efficacy of SGLT2 inhibitors and certain baseline patient parameters (Table 6). There was a negative correlation between LVEF and the efficacy of SGLT2 inhibitors (rho = −0.270, *P* < 0.001), indicating that lower baseline LVEF was associated with greater treatment efficacy. Conversely, NT-proBNP levels showed a positive correlation with treatment efficacy (rho = 0.232, *P =* 0.002), suggesting that higher baseline NT-proBNP levels correlate with better responses to SGLT2 inhibitors. These findings underscore the importance of considering baseline cardiac function and stress markers when evaluating patient suitability for SGLT2 inhibitor therapy in managing HF post- AMI.

**TABLE 6:** Correlation analysis between SGLT2 inhibitors efficacy and various factors.

Parameters	rho	P
LVEF (%)	−0.270	<0.001
NT-proBNP (pg/mL)	0.232	0.002

SGLT2, sodium-dependent glucose transporters 2; LVEF, left ventricular ejection fraction; NT-proBNP, N-terminal prohormone of brain natriuretic peptide.

### 3.9 Multivariate logistic regression analysis

The multivariate logistic regression analysis identified independent risk factors of SGLT2 inhibitor efficacy in treating HF post- AMI (Table 7). Lower LVEF was associated with improved treatment outcomes, as indicated by an odds ratio (OR) of 0.841 (95% CI, 0.755–0.937; *P =* 0.002), highlighting its inverse relationship with treatment efficacy. Higher NT-proBNP levels were also positively correlated with treatment success, with an OR of 1.007 (95% CI, 1.001–1.013; *P =* 0.012), suggesting that elevated baseline NT-proBNP slightly increases the likelihood of a positive response to SGLT2 inhibitors. These findings emphasize the role of baseline cardiac function and biomarker levels in predicting the therapeutic benefits of SGLT2 inhibitors in this patient population.

**TABLE 7:** Multivariate logistic regression analysis.

Parameters	Std error	Wald stat	OR	95% CI	P
LVEF (%)	0.055	−3.141	0.841	0.755–0.937	0.002
NT-proBNP (pg/mL)	0.003	2.511	1.007	1.001–1.001	0.012

SGLT2, sodium-dependent glucose transporters 2; LVEF, left ventricular ejection fraction; NT-proBNP, N-terminal prohormone of brain natriuretic peptide; OR, odds ratio; CI, confidence interval.

## 4 Discussion

In this study, SGLT2 inhibitors were associated with improved in improving cardiac function following AMI in patients with HF. SGLT2 inhibitors have emerged as a promising class of drugs in cardiology, primarily due to their ability to modulate glucose and sodium homeostasis (Liang et al., 2022). In our study, these agents significantly improved LVEF and reduced markers of cardiac stress such as NT-proBNP, troponin I, and hs-CRP compared to conventional treatment. These findings align with existing literature, suggesting a pivotal role of SGLT2 inhibitors in modifying cardiac load and metabolic demands (Lin et al., 2022). Importantly, the benefits of SGLT2 inhibitors were observed on top of a guideline-directed therapy including ARNI, highlighting their additive value in the comprehensive management of post-AMI HF. By reducing plasma glucose and sodium reabsorption in the renal proximal tubules, SGLT2 inhibitors facilitate osmotic diuresis and natriuresis, thereby decreasing preload and afterload (Lioud et al., 2022). This mechanism was particularly beneficial post-AMI, where managing volume status and myocardial strain was crucial (Mascolo et al., 2022).

The metabolic modulation by SGLT2 inhibitors extends beyond glucose control, affecting myocardial energy substrate utilization (Morillas et al., 2022). AMI invariably leads to myocardial energy crisis, where the heart predominantly relies on glucose metabolism due to ischemic conditions (Oriecuia et al., 2024). SGLT2 inhibitors shift the myocardial substrate preference from glucose to ketone bodies, which were more energy-efficient fuels (Rosano et al., 2021). This metabolic shift not only meets the energy demands of a stressed myocardium but also curtails the deleterious effects of elevated glucose oxidation rates seen in HF (Sarzani et al., 2020). In the specific context of post-AMI, where an acute energy crisis occurs in the infarcted and border zones, this provision of an efficient alternative fuel source may be particularly crucial for salvaging jeopardized myocardium and supporting contractile function (Xiong et al., 2024). Such alterations in myocardial metabolism might explain the improved cardiac function observed in the SGLT2 group compared to conventional therapy.

Our results also highlight the potential anti-inflammatory and antifibrotic benefits of SGLT2 inhibitors, inferred from the decreased hs-CRP levels in the SGLT2 group. Inflammation was a critical component of cardiac remodeling post-AMI, perpetuating myocardial injury and ventricular impairment (Roe, 2021). The reduction in inflammatory markers suggests that SGLT2 inhibitors might mitigate cardiac remodeling processes, blunting the inflammatory cascade that contributes to adverse cardiac outcomes (Zeng et al., 2021). This anti-inflammatory effect could be attributed to the systemic metabolic improvements and the resultant reduction in oxidative stress (Yurista et al., 2020). Following AMI, a robust inflammatory response drives maladaptive remodeling; thus, mitigating this inflammation with SGLT2 inhibitors could directly translate to an attenuation of adverse ventricular remodeling and a preservation of ventricular geometry (Yoo et al., 2025).

Another key finding was the statistically significant decrease in angina pectoris in the SGLT2 group. By optimizing cardiac hemodynamics and improving myocardial metabolism, SGLT2 inhibitors may enhance cardiac efficiency, reducing the frequency of ischemic episodes and angina in vulnerable post-AMI populations (Yu et al., 2021). Coupled with their diuretic effects, these agents potentially offer comprehensive cardiovascular benefits by improving oxygen supply-demand balance within the ischemic myocardium. This may be attributed to improved myocardial oxygen utilization due to a shift in energy substrate preference (e.g., towards ketone bodies) and potential direct anti-ischemic effects, which collectively could alleviate myocardial ischemia and its symptomatic manifestation as angina (Werkman et al., 2023). In post-AMI patients, who often have residual coronary disease and microvascular dysfunction, this optimized hemodynamic profile and metabolic efficiency are especially beneficial for reducing recurrent ischemic episodes (Ci Mee et al., 2025).

Our analysis identified baseline LVEF and NT-proBNP levels as significant predictors of treatment response, underscoring the clinical relevance of baseline cardiac function in SGLT2 therapeutic efficacy. The lower baseline LVEF was associated with better treatment outcomes, indicating greater potential for functional recovery in severely impaired hearts. This aligns with the notion that patients with advanced cardiac dysfunction may derive amplified benefits from therapies that enhance myocardial performance and reduce cardiac loading. Conversely, higher baseline NT-proBNP levels, indicative of significant cardiac stress and volume overload, correlated positively with treatment efficacy, suggesting that SGLT2-mediated volume reduction and cardiac unloading were particularly advantageous in substantially burdened myocardium. While these clinical markers offer valuable prognostic insight, the underlying biological mechanisms for this differential response remain incompletely understood. Future mechanistic studies, potentially involving advanced imaging, myocardial biopsies, or molecular profiling in targeted cohorts, could validate these predictors and elucidate the pathways governing treatment efficacy and resistance.

Our findings of significant improvements in LVEF and reductions in NT-proBNP extend the established benefits of SGLT2 inhibitors from large-scale outcome trials to the specific post-AMI HF population. Landmark trials such as DAPA-HF and EMPEROR-Reduced, which primarily demonstrated robust reductions in heart failure hospitalizations and cardiovascular mortality in a broad HFrEF cohort (Gonzalez-Franco et al., 2024), provide the foundational evidence for this drug class. While our single-center study was not powered to assess those hard clinical endpoints, the consistent and pronounced improvements we observed in cardiac structure and function (LVEF, LV volumes) and cardiac stress (NT-proBNP) align with the proposed mechanisms—such as reverse remodeling and reduced ventricular load—that underpin the clinical benefits seen in those trials. This suggests that the cardioprotective effects of SGLT2 inhibitors are also operative and measurable in the high-risk context of recent myocardial infarction.

One of the primary limitations of this study was its retrospective and single-center nature, which inherently subjects the findings to potential confounding factors and biases despite well-matched baseline characteristics. All participants were recruited from a single center in China, which may limit the extrapolation of our findings to other ethnicities or healthcare settings. Additionally, the non-randomized assignment of treatments may introduce selection bias, as physicians’ decisions could be influenced by unmeasured patient characteristics. Although baseline characteristics were well-balanced, this potential source of bias should be considered. Finally, our study lacked subgroup analyses based on important variables such as diabetes status, renal function, and age. Therefore, the consistency of the treatment effect across these different patient profiles could not be evaluated and warrants investigation in future studies. Future prospective trials with randomized designs and multi-center, multi-ethnic populations could provide more definitive insights into the causal relationships and mechanistic pathways by which SGLT2 inhibitors exert their cardioprotective effects post-AMI. Additionally, exploring the role of SGLT2 inhibitors across different phenotypes of HF and varying degrees of myocardial injury could offer valuable perspectives on their therapeutic scope.

While SGLT2 inhibitors present as a transformative approach in managing post-AMI HF, they were not devoid of adverse effects. Our findings noted comparable incidences of adverse reactions like nausea and dizziness between the groups, though with a reduction in the incidence of angina pectoris in the SGLT2 group. This suggests a favorable safety profile, albeit necessitating vigilance to monitor for potential renal effects or electrolyte disturbances, given their impact on renal function and glucose metabolism. While our 3-month data showed no significant renal impairment or electrolyte imbalances, the long-term effects and optimal monitoring strategies for renal function and electrolytes in post-AMI HF patients receiving SGLT2 inhibitors remain important considerations for future studies with extended follow-up. Individual patient risk profiles and comorbid conditions should guide clinical decision-making to optimize the therapeutic window while minimizing potential harms.

Furthermore, the potential applicability of our findings to other cardiovascular phenotypes warrants exploration. While our study focused on HF post-AMI—a context often associated with reduced ejection fraction—it remains to be investigated whether similar benefits would be observed in patients with HFpEF or those experiencing recurrent AMI, as the underlying pathophysiologies and hemodynamic profiles differ. Future studies are needed to validate the efficacy of SGLT2 inhibitors across this broader spectrum of cardiovascular patients.

## 5 Conclusion

In conclusion, the study suggests the potential of SGLT2 inhibitors as beneficial agents in improving cardiac outcomes following AMI, attributable to their multifaceted mechanism of action encompassing diuretic, antihypertrophic, anti-inflammatory, and metabolic modulation. These findings not only reinforce their inclusion in the therapeutic armamentarium for managing HF post-AMI but also stimulate further inquiry into their full clinical potential and underlying mechanistic pathways. A nuanced understanding of individual patient characteristics, such as cardiac function markers and the extent of myocardial strain, was crucial in tailoring SGLT2 therapy and augmenting its benefits in diverse cardiac populations. As the landscape of cardiovascular therapeutics evolves, the role of SGLT2 inhibitorsundoubtedly continue to expand, necessitating ongoing research to unravel their impact on cardiac health and long-term outcomes.

## Data Availability

The raw data supporting the conclusions of this article will be made available by the authors, without undue reservation.
